# Supplementary material for: Follistatin Is a Novel Chemoattractant for Migration and Invasion of Placental Trophoblasts of Mice
Source: Cells. 2022 Nov 29;11(23):3816. doi: 10.3390/cells11233816 (PMC9741044; doi:10.3390/cells11233816)
Supplement: Supplementary file 1 [file cells-11-03816-s001.zip › cells-1949370-supplementary.pdf]

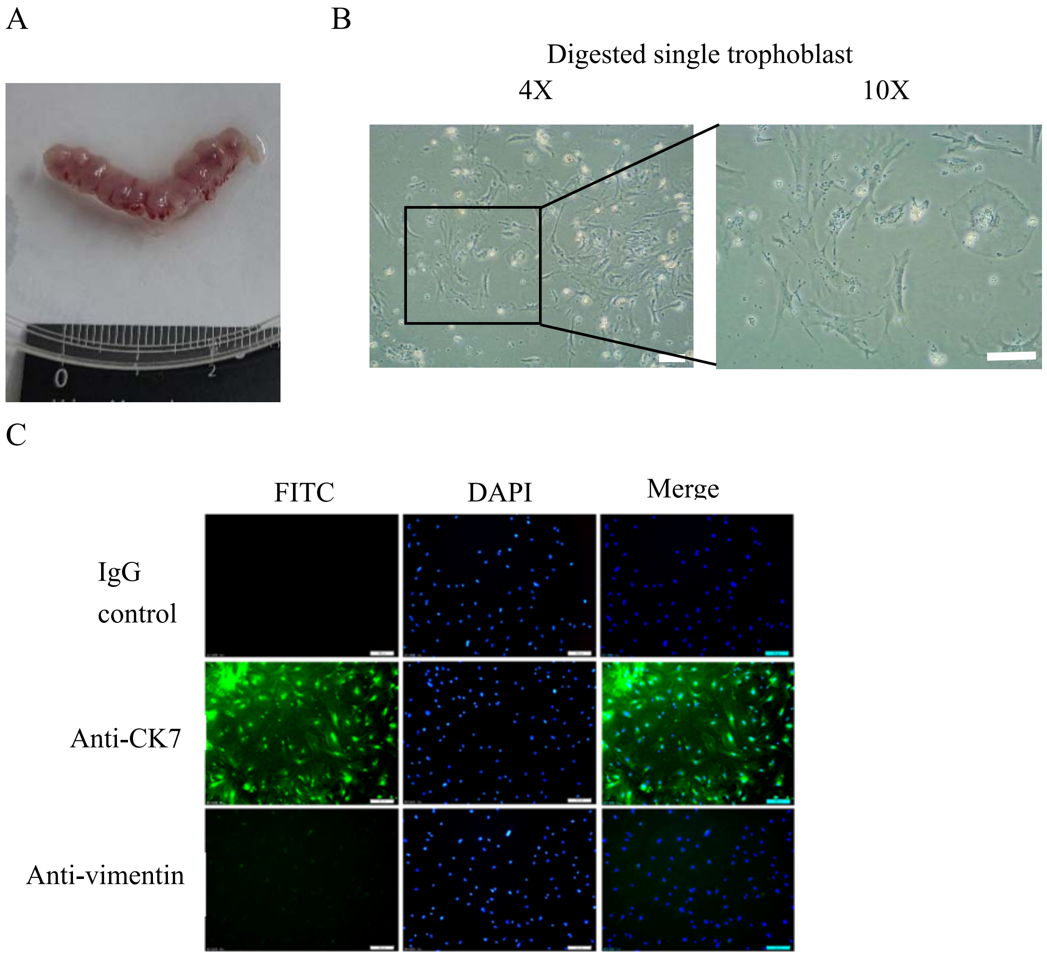

Figure S1. The origination and identification of primary cultured trophoblasts of embryonic day 8.5 (E8.5) mouse. (A) the uterus of embryonic day 8.5 (E8.5) mouse. The isolated placental tissue was digested and filtered twice. (B) Digested placental thophoblast of mouse spreaded in a petri dish with 10%-FBS-DMEM-F12 at $37^{\circ}C$ contained 5% CO_2, respectively. (C) Trophoblast cells in which more than 95% of cells were positive for expression of cytokeratin-7 (CK7) as trophoblast marker. Scale bar = 100 μm.

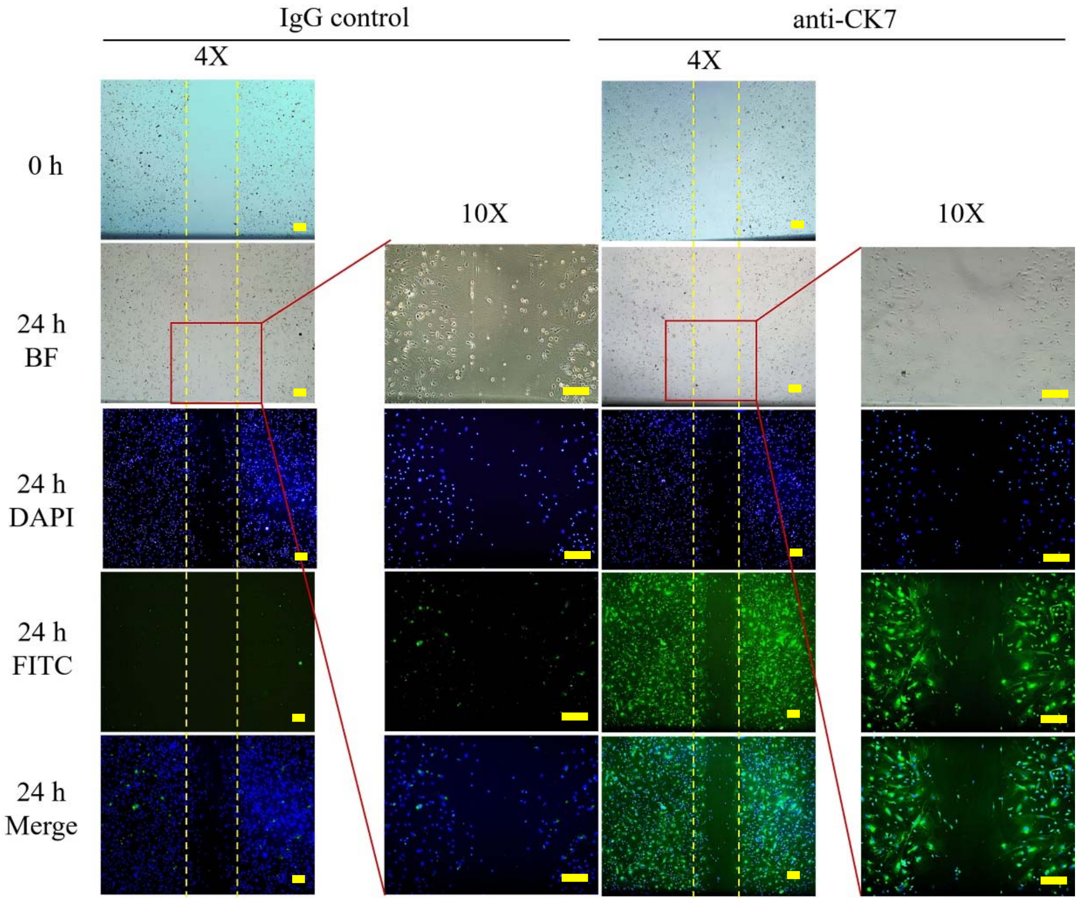

Figure S2. The migratory cells in the wound healing assay were also stained with cytokeratin-7 (CK7), which confirmed that the migratory cells in the wound healing assay are indeed trophoblast cells expressed CK7 positively. Scale bar = 100 μm.
